# In-hospital deep vein thrombosis after tibial plateau fractures: incidence, laterality/anatomy, and risk factors in a multicenter retrospective cohort of 3366 patients

**DOI:** 10.1186/s10195-026-00916-8

**Published:** 2026-03-26

**Authors:** Shuo Yang, Guoqiang Li, Yiran Li, Yubin Long, Jiaqi Zhang, Lingfeng Liu, Zihang Zhao, Changsheng Sun, Lin Liu, Lin Jin, Tao Wang, Zhiyong Hou

**Affiliations:** 1 Department of Orthopaedic Surgery, The Third Hospital of Hebei Medical University, 139 Ziqiang Road, Shijiazhuang, 050051 Hebei People’s Republic of China; 2 Orthopaedic Research Institute of Hebei Province, Shijiazhuang, Hebei People’s Republic of China; 3 The Third Department of Orthopedics, Baoding First Central Hospital, Hebei, People’s Republic of China; 4 Department of Anesthesiology, The Third Hospital of Hebei Medical University, Shijiazhuang, Hebei People’s Republic of China; 5 NHC Key Laboratory of Intelligent Orthopaedic Equipment (The Third Hospital of Hebei Medical University), Shijiazhuang, Hebei People’s Republic of China; 6 Engineering Research Center of Orthopedic Minimally Invasive Intelligent Equipment, China Ministry of Education, Shijiazhuang, China; 7 Key Laboratory of Biomechanics of Hebei Province, Shijiazhuang, Hebei China; 8 Key Laboratory of Precise Assessment, Diagnosis, and Treament of Soft Tissue Injury of Hebei Province, Shijiazhuang, Hebei China

**Keywords:** Tibial plateau fractures, Venous thrombosis, Risk factors, Retrospective studies

## Abstract

**Background:**

We aimed to determine the cumulative in-hospital incidence, anatomic distribution, and predictors of duplex ultrasonography-detected lower-extremity deep vein thrombosis (DVT) in patients hospitalized with tibial plateau fractures under routine thromboprophylaxis and protocolized surveillance.

**Methods:**

We retrospectively analyzed consecutive adults (≥ 18 years) admitted to two level-I trauma centers (November 2014–January 2024). Bilateral duplex ultrasonography was performed on admission/preoperatively (as soon as feasible after arrival and prior to surgery) and repeated serially during hospitalization (approximately every 5–7 days and when clinically suspected). The primary outcome was cumulative in-hospital DVT from admission to discharge; isolated intermuscular calf-muscle vein thrombosis (soleal/gastrocnemius) was recorded descriptively but excluded from the primary endpoint. Multiple injuries (polytrauma) were defined as ≥ 1 additional acute traumatic lesion beyond the index tibial plateau fracture documented on admission. Pulmonary embolism (PE) was assessed only when clinically suspected and confirmed by computed tomography pulmonary angiography (CTPA). Multivariable logistic regression identified independent predictors; receiver operating characteristic (ROC) analysis evaluated discrimination for key continuous predictors.

**Results:**

Among 3366 patients, 675 developed in-hospital DVT (20.0%). First detection occurred preoperatively in 432 (64.0%) patients and postoperatively in 243 (36.0%). Most events were distal (584/675, 86.5%) and ipsilateral (617/675, 91.4%); proximal DVT occurred in 91 patients (2.7% of the cohort). Independent predictors included age, multiple injuries, anemia, alcohol use, and inverse associations with activated partial thromboplastin time and serum sodium. ROC analysis of age showed modest discrimination (area under the curve [AUC] 0.593) with a cohort-derived Youden threshold of 42 years (age ≥ 42 years). Symptomatic PE occurred in five patients (0.15% of the cohort) and was confirmed by CTPA.

**Conclusions:**

Under protocolized inpatient duplex surveillance, patients with tibial plateau fracture had a substantial in-hospital burden of predominantly distal and ipsilateral DVT. Increasing age was independently associated with DVT; however, age alone showed limited discriminatory ability (AUC 0.593), with a cohort-derived Youden threshold of 42 years (age ≥ 42 years). Multiple injuries, anemia, alcohol use, shorter activated partial thromboplastin time (APTT), and lower serum sodium were independently associated with DVT, while the discrimination of age alone was modest. These findings may help inform risk stratification and the consideration of systematic or risk-adapted surveillance and individualized perioperative management; prospective studies are needed to determine whether risk-tailored strategies improve clinically meaningful outcomes.

## Introduction

Lower-extremity deep vein thrombosis (DVT), a major manifestation of venous thromboembolism (VTE), is a major cause of preventable morbidity and mortality after trauma [1]. In orthopedic patients, DVT may progress to pulmonary embolism (PE), prolong hospitalization, and lead to post-thrombotic syndrome with lasting functional impairment. Tibial plateau fractures are complex intra-articular injuries—often due to high-energy mechanisms—managed initially with limb immobilization and frequently staged surgery; together with endothelial injury and trauma-driven inflammatory and coagulation activation, these factors fulfill Virchow’s triad (stasis, endothelial injury, hypercoagulability) and create a prothrombotic milieu [2]. Despite routine chemoprophylaxis, VTE has not been eliminated in orthopedic trauma cohorts [2].

The reported incidence of DVT after tibial plateau fractures varies widely across studies, largely because surveillance strategies and case definitions differ substantially. DVT may be symptomatic, leading to imaging triggered by clinical suspicion, or asymptomatic and detected only through routine screening. Consequently, symptom-driven diagnostic pathways generally report lower incidences, whereas systematic duplex ultrasonography—especially when calf veins are routinely assessed—reveals a higher overall burden that includes clinically silent events [1, 3, 4]. Across cohorts, rates range from approximately 5% to > 50%, influenced by scan timing and frequency, as well as whether isolated calf-muscle (intermuscular) vein thromboses are counted in addition to proximal/distal deep-vein events [1, 3, 4]. Notably, trauma-induced hypercoagulability can develop rapidly, and a substantial proportion of thrombi may be detectable within 48 h after admission [5]. Because the detected incidence of DVT depends strongly on when and how often ultrasound surveillance is performed during hospitalization, a standardized screening schedule is essential for capturing the overall in-hospital DVT burden.

Risk determinants are multifactorial. Across lower-extremity fractures, increasing age, higher injury severity, immobility, and comorbidities recur as general risk factors [6–8]. Within tibial plateau fractures specifically, prior work has identified male sex, hypertension, open injury, and hyponatremia as independent correlates of preoperative DVT [4], whereas postoperative DVT has been linked to age ≥ 41 years, general (versus regional) anesthesia, hyponatremia, longer operative time, and elevated D-dimer [3]. However, many existing studies are limited by modest sample sizes, single time-point assessments, symptom-triggered imaging, or incomplete interrogation of calf venous territories. In contrast, our analysis leverages a large, multicenter cohort and a protocolized surveillance strategy with serial bilateral duplex ultrasonography (on admission/preoperatively and repeated during hospitalization), enabling capture of both clinically apparent and clinically silent events. We further characterize the anatomic distribution of DVT at the vein-segment level (e.g., proximal versus infrapopliteal deep veins and laterality) on the basis of comprehensive segment interrogation. Finally, beyond multivariable modeling of risk factors, we assess the discriminative performance of a key continuous predictor using ROC analysis to provide an empirically derived threshold for risk stratification. Thus, a comprehensive, large-sample analysis with systematic screening is needed to clarify the true in-hospital DVT burden and associated risk factors in patients with tibial plateau fractures.

This study aimed to conducted a large, multicenter retrospective analysis of consecutive tibial plateau fracture inpatients managed with routine thromboprophylaxis and protocolized bilateral duplex ultrasonography performed on admission (as soon as feasible after arrival) and repeated serially during hospitalization, to estimate the overall in-hospital DVT incidence and identify associated risk factors. Our objectives were to: (1) establish the in-hospital incidence of DVT; (2) delineate laterality (injured versus contralateral limb) and anatomic distribution (distal versus proximal; specific segments) of thrombi; (3) identify independent risk factors spanning demographics, injury features, comorbidities, and admission laboratory data; and (4) among continuous predictors retained as independent factors, to evaluate ROC discrimination and derive pragmatic cutoffs for risk stratification (e.g., age). We hypothesized that DVT would be common despite prophylaxis, predominantly distal and ipsilateral, and associated with increasing age and greater injury burden. We also explored anemia, alcohol history, and laboratory indicators reflecting a hypercoagulable or stress milieu as additional candidate predictors.

## Methods

### Ethics statement

This retrospective review analyzed electronic medical records of patients with tibial plateau fractures treated at two level-I tertiary trauma centers between November 2014 and January 2024. The study protocol was approved by the institutional review boards of both institutions prior to data extraction and complied with the ethical principles of the Declaration of Helsinki (approval nos. 2025–373-1 and 2,024,181). Data extraction for research purposes was performed on 1 September 2025. Given the retrospective design and use of deidentified data, the requirement for informed consent was waived. Throughout data handling, investigators had no access to direct patient identifiers.

### Study design and patients

We conducted a retrospective cohort study of consecutive adults (≥ 18 years) admitted with tibial plateau fractures to Hebei Medical University Third Hospital and Baoding No. 1 Central Hospital from November 2014 to January 2024. Both hospitals are level-I tertiary trauma centers where, during the study period, bilateral lower-extremity duplex ultrasonography was routinely used to screen major orthopedic injuries. Patients were identified from the orthopedic trauma registry and the electronic medical record.

Inclusion criteria were: age ≥ 18 years; imaging-confirmed acute tibial plateau fracture; and hospitalization for operative or non-operative management. Exclusion criteria were: pathological fractures (e.g., metastasis); old fractures (> 3 weeks from injury); documented history of DVT or pulmonary embolism; or current therapeutic anticoagulation for other indications.

**Fig. 1:**
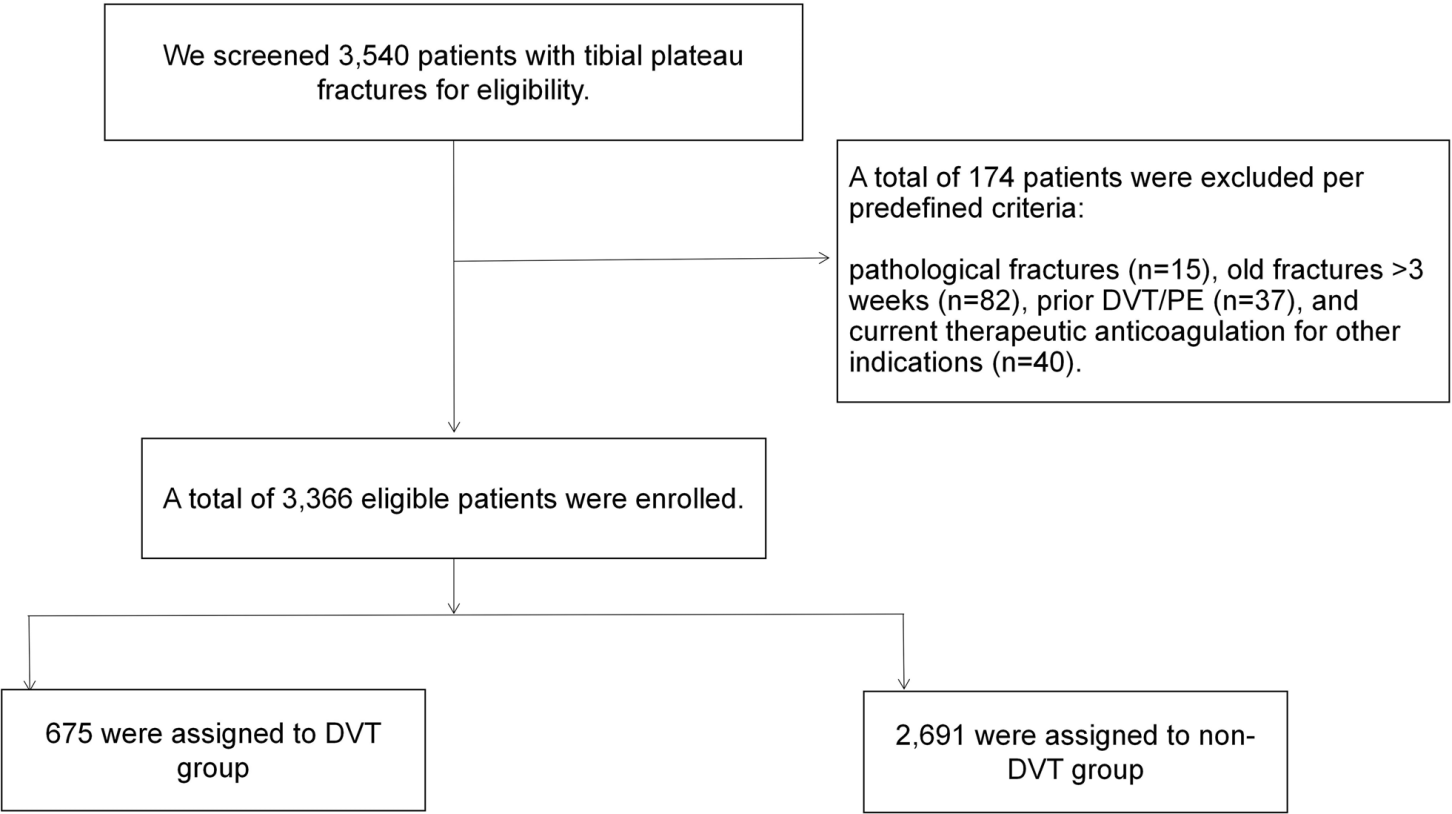
Exclusion criteria and the final cohort of eligible patients included in the study

Using these criteria, we included 3366 patients (2105 men; 1261 women), who were subsequently categorized into DVT and non-DVT groups on the basis of ultrasonographic diagnosis during the index hospitalization (Fig. 1).

### DVT definitions and classification

The primary outcome was cumulative in-hospital DVT, defined as any lower-extremity DVT detected by duplex ultrasonography from admission to discharge, including DVT identified on the initial admission/preoperative scan and those detected on subsequent inpatient surveillance scans. For the primary analysis, we did not distinguish admission-detected versus later-detected DVT; all detected events contributed to the cumulative in-hospital incidence.

Because duplex ultrasonography was performed routinely irrespective of symptoms, we did not categorize DVT as symptomatic versus asymptomatic; all ultrasound-detected DVT events were analyzed together in the primary analysis.

For the primary (patient-level) analysis, DVT was classified hierarchically by the most proximal venous segment detected in either limb:

Proximal DVT was defined as thrombosis at or above the popliteal vein (popliteal, superficial/common femoral, profunda femoris, iliac).

Distal DVT was defined as thrombosis confined to infrapopliteal deep veins (anterior tibial, posterior tibial, peroneal).

Per protocol, isolated intermuscular (soleal/gastrocnemius) vein thromboses were not counted as DVT in the primary analysis but were recorded descriptively. This approach was chosen to improve comparability with studies that define DVT as thrombosis of named deep veins, because isolated calf-muscle vein thrombosis is variably classified across literature [1, 4, 15, 16]. For completeness, we also noted “mixed involvement” (concomitant proximal and distal thrombosis within the same limb), but mixed cases were assigned to the proximal category for the main incidence comparison to keep totals consistent with the two-level reporting (e.g., proximal 91 [13.5%] versus distal 584 [86.5%] among DVT-positive patients).

### DVT prophylaxis and detection

On admission, patients without contraindications (e.g., active bleeding or planned surgery within 12 h) received pharmacologic prophylaxis with low-molecular-weight heparin (LMWH; enoxaparin 40 mg [≈4000 anti-Xa IU] subcutaneous [SC] once daily) plus limb elevation; dosing was adjusted for renal impairment and body weight per institutional protocol. LMWH was withheld approximately 12 h before surgery and restarted 12–24 h after wound closure once hemostasis was secured. Patients with polytrauma followed the same thromboprophylaxis protocol. LMWH was administered unless contraindicated; initiation could be deferred in the setting of active/unstable bleeding or other high bleeding risk, and for staged procedures, LMWH was temporarily interrupted per the perioperative schedule and restarted once hemostasis was secured.

Mechanical prophylaxis (intermittent pneumatic compression) was applied only to limbs without injury and without known/suspected DVT—typically the contralateral, uninjured limb—and was not used on any limb with established DVT or when soft tissue status/fixation rendered compression inappropriate. In patients with bilateral lower limb injuries or DVT, mechanical prophylaxis was withheld. This regimen reflects our centers’ protocol for VTE prevention in orthopedic trauma.

All patients underwent initial bilateral lower-extremity duplex ultrasonography as soon as feasible after hospital admission and before any surgical intervention. The standardized surveillance workflow was as follows: injury → hospital admission (with the injury-to-admission interval recorded) → initial bilateral duplex ultrasonography → subsequent inpatient surveillance scans approximately every 5–7 days, as well as whenever DVT was clinically suspected, until discharge. The 5–7-day interval was the protocol target for all patients (including polytrauma), although the exact timing could be adjusted on the basis of clinical condition and feasibility.

For descriptive analyses, the timing of first DVT detection was categorized as preoperative or postoperative according to whether the first positive duplex ultrasonography occurred before or after definitive fracture surgery. Experienced vascular sonographers assessed the common femoral, superficial femoral, profunda femoris, popliteal, anterior tibial, posterior tibial, and peroneal veins. DVT was diagnosed by non-compressibility under probe pressure, direct visualization of intraluminal thrombus, or absent flow on Doppler augmentation. For classification, popliteal or more proximal thrombosis (femoral/iliac) was defined as proximal DVT; clots confined to infrapopliteal deep veins (tibial/peroneal) were considered distal DVTs. Superficial venous thrombosis and isolated intermuscular (soleal/gastrocnemius) vein thromboses were not counted as DVTs in the primary analysis. If both distal and proximal DVTs were present, the case was categorized as proximal for incidence reporting.

When a DVT was detected, patients were managed according to contemporary evidence-based guidelines [9, 10]. Anticoagulation was the mainstay of therapy; inferior vena cava (IVC) filters were not used routinely, and were considered only when anticoagulation was contraindicated or had to be temporarily withheld owing to high bleeding risk, with planned retrieval as soon as feasible. These principles align with the CHEST 2021 and ASH 2020 recommendations (e.g., against routine filter use in anticoagulated VTE; for filter placement when there is an absolute contraindication to anticoagulation).

In general, proximal DVT or extensive thrombosis in a patient who could not receive anticoagulation around urgent fracture surgery prompted multidisciplinary team (MDT) review to weigh temporary IVC filter placement versus intensified perioperative anticoagulation and timing of surgery, consistent with guideline frameworks. Distal DVTs were treated with therapeutic anticoagulation—typically enoxaparin 1 mg/kg SC every 12 h (q12 h) (≈100 anti-Xa IU/kg q12 h)—with clinical and ultrasonographic surveillance; filters were reserved for exceptional high-risk scenarios when anticoagulation was not possible. In our series, 86 patients (12.7% of DVT cases) received a temporary filter after MDT assessment; retrieval was scheduled once anticoagulation could be safely instituted. PE was assessed only when clinically suspected. Computed tomography pulmonary angiography (CTPA) was used for confirmation, and only imaging-confirmed symptomatic PE events were recorded.

### Data collection

We extracted data on a range of potential risk factors for DVT on the basis of prior studies and clinical plausibility. Demographics included age and sex. Injury characteristics included (i) mechanism severity grade coded ordinally as grade 1 (low-energy)—ground-level or same-level events (ground-level fall, slip, trip, standing/walking fall); grade 2 (intermediate-energy)—low-height falls or similar (fall from low height, fall on stairs/steps, bicycle, skateboard); and grade 3 (high-energy)—traffic/motor-vehicle related (car crash, motorcycle, electric bike/electric vehicle [EV], other traffic collisions); (ii) multiple injuries (yes/no), defined as ≥ 1 additional acute traumatic lesion beyond the index tibial plateau fracture documented on admission (e.g., other limb or axial fractures; head/neck injury, including intracranial hemorrhage; thoracic/abdominal/retroperitoneal injury; major soft tissue injury or compartment syndrome; limb neurovascular injury); diagnoses representing nontraumatic comorbidities (e.g., hypertension/diabetes) and thromboembolic events (DVT/PE) did not qualify (established injury severity scores such as the Injury Severity Score [ISS]/Abbreviated Injury Scale [AIS] were not routinely available in the registry/medical records; therefore, we used this pragmatic definition to operationalize polytrauma for analysis); (iii) open fracture (yes/no); and (iv) injury-to-admission interval (days), defined as the number of days between the recorded time/date of injury and hospital admission. Lifestyle factors included current smoking and alcohol use history (regular consumption defined as habitual drinking at least weekly). Comorbidities captured on admission were hypertension, diabetes mellitus, coronary heart disease, and prior cerebral infarction (stroke). Admission laboratory-based conditions were defined a priori: anemia (hemoglobin < 120 g/L in female individuals or < 130 g/L in male individuals), hypoproteinemia (serum albumin < 35 g/L), and electrolyte disturbances, including hyponatremia (serum Na < 135 mmol/L) and hypokalemia (K < 3.5 mmol/L). All variables were abstracted from the trauma registry and electronic medical records using uniform case-report templates.

A comprehensive laboratory panel was obtained from the first blood draw on hospital admission as part of routine trauma evaluation. Blood sampling was performed prior to surgical intervention whenever feasible and before or at the initiation of pharmacologic thromboprophylaxis. This included (i) a full hematology profile (complete blood count [CBC] with five-part differential; red-cell indices such as mean corpuscular volume [MCV], mean corpuscular hemoglobin [MCH], mean corpuscular hemoglobin concentration [MCHC], red cell distribution width [RDW]; and platelet indices, including platelet count [PLT], mean platelet volume [MPV], platelet distribution width [PDW], plateletcrit [PCT]); (ii) serum biochemistry (electrolytes Na^+^, K^+^, Cl^−^,Ca^2+^ and Mg^2+^; renal function urea/creatinine; glucose; liver profile tests such as alanine aminotransferase [ALT], aspartate aminotransferase [AST], alkaline phosphatase [ALP], gamma-glutamyl transferase [GGT], bilirubin tests, including total bilirubin [TBIL] and direct bilirubin [DBIL]; proteins albumin [ALB], total protein [TP], globulin [GLOB]; lipid panel tests such as total cholesterol [TC], triglycerides [TG], high-density lipoprotein cholesterol [HDL-C], low-density lipoprotein cholesterol [LDL-C]; calculated anion gap and osmolality); (iii) tissue-injury/metabolic enzymes (lactate dehydrogenase [LDH], hydroxybutyrate dehydrogenase [HBDH], creatine kinase [CK], CK-MB); and (iv) a coagulation–fibrinolysis panel (prothrombin time [PT], international normalized ratio [INR], activated partial thromboplastin time [APTT] and ratio, thrombin time [TT] and ratio, fibrinogen, D-dimer, fibrin/fibrinogen degradation products [FDP], antithrombin III). In total, each patient contributed dozens of laboratory variables, reflecting the breadth of physiological domains evaluated. All assays were performed by the hospital central laboratories using standardized methods with internal quality control. These extensive laboratory data were collected to explore any associations between baseline coagulation/inflammatory status and DVT development, as suggested by prior investigations [11].

The anatomical distribution of DVT (when present) was determined from ultrasound reports. We noted whether the thrombus was in the injured leg, the uninjured leg, or bilateral; and the specific veins involved. For summary purposes, clots were tallied by vein segment (e.g., number of patients with peroneal vein thrombosis, etc.) to characterize which venous segments were most often affected in this cohort.

### Statistical analysis

Patients were divided into two groups—DVT group and non-DVT group—on the basis of whether any DVT was detected during their hospital course. We first performed univariate comparisons between the groups. For continuous variables, normality was assessed; variables with approximately normal distribution are presented as mean ± standard deviation and were compared with the *t*-test, while non-normally distributed variables are presented as median with interquartile range (IQR) and compared with the Mann–Whitney *U* test. Categorical variables are summarized as counts and percentages and were compared using the chi-squared test or Fisher’s exact test, as appropriate. A two-tailed *p* < 0.05 was considered statistically significant for these univariate tests.

To identify independent risk factors for DVT, we carried out a binary logistic regression analysis. Candidate variables for the model included those with a univariate *p*-value < 0.05. Given the large number of laboratory variables, we avoided collinearity by selecting one representative from highly correlated sets (for example, hemoglobin was chosen to represent anemia status rather than including both hemoglobin and hematocrit). The regression model used a backward stepwise elimination (likelihood ratio) procedure to retain significant predictors. Results are reported as odds ratios (ORs) with 95% confidence intervals (CIs) and *p*-values.

Finally, for the continuous independent predictors identified, we constructed ROC curves to evaluate their ability to distinguish DVT versus non-DVT patients. For each, we calculated the area under the curve (AUC) with 95% CI. We determined an “optimal” cutoff value that maximized the Youden index (sensitivity + specificity − 1). Sensitivity and specificity at these cutoffs were computed. All statistical analyses were performed using SPSS version 26.0 (IBM Corp., Armonk, NY, USA) and MedCalc version 20.0.

## Results

### Patient characteristics and DVT incidence

**Fig. 2:**
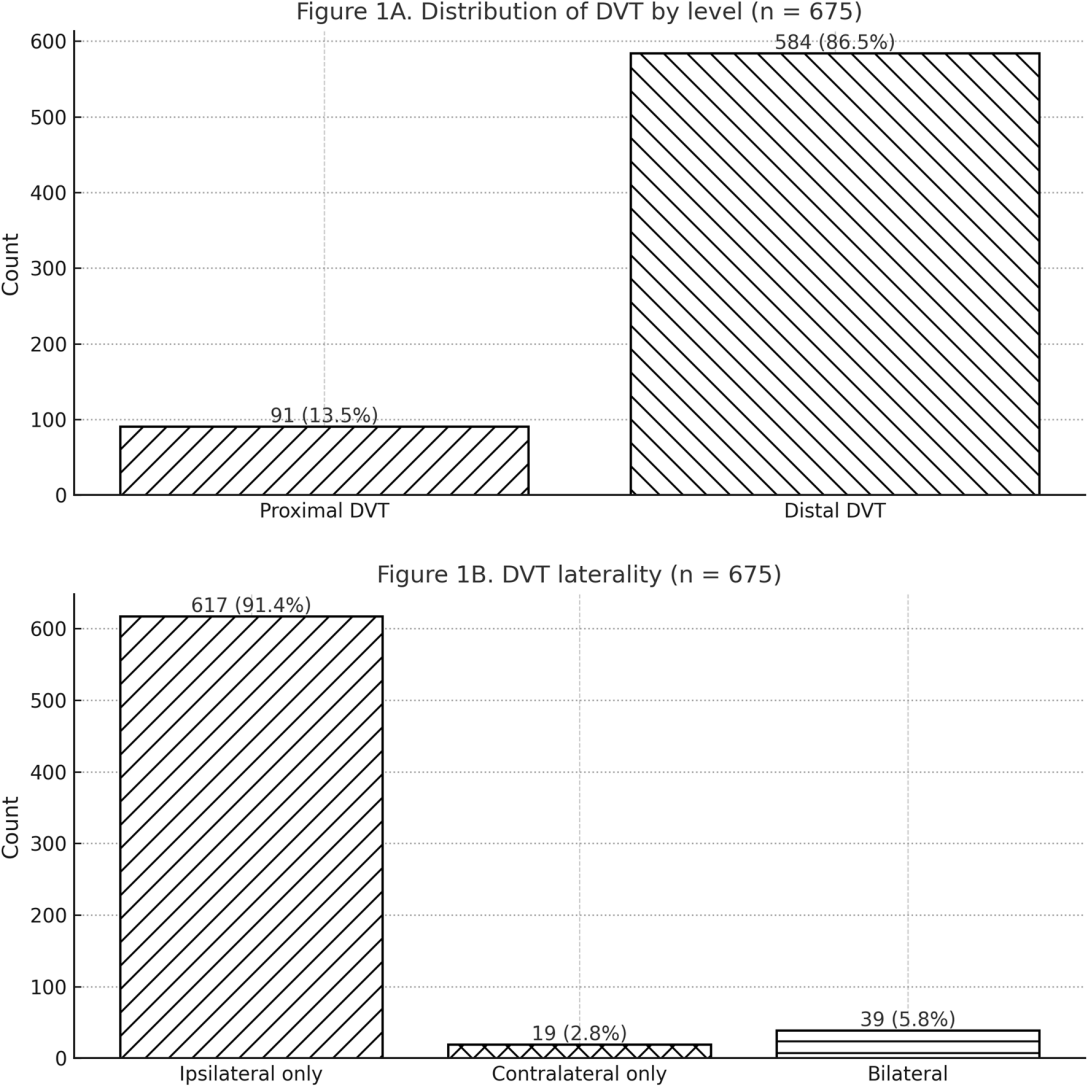
Distribution of DVT by level and DVT distribution

A total of 3366 patients with tibial plateau fractures met the inclusion criteria. The cohort had a median age of 46 years (IQR 35–56) and included 2105 male (62.5%) and 1261 female (37.5%) patients. Overall, 675 patients were diagnosed with DVT during hospitalization, corresponding to an incidence of 20.0%. Among the 675 DVT-positive patients, the first DVT was detected preoperatively in 432 patients (64.0%) and postoperatively in 243 patients (36.0%), based on the timing of the first positive duplex ultrasonography relative to definitive fracture surgery. Of these, 91 patients (13.5% of DVT cases; 2.7% of the entire cohort) had a proximal DVT involving the popliteal or more proximal veins, whereas the remaining 584 patients (86.5%) had distal DVT confined to the infrapopliteal deep veins. Thus, distal thrombi constituted the great majority of events. Notably, in 617 DVT patients (91.4%), the thrombus was located only in the leg on the side of the fracture (“ipsilateral” DVT). A total of 19 patients (2.8% of DVT cases) had DVT only in the contralateral (uninjured) leg, and 39 patients (5.8%) had bilateral DVT. Bilateral or contralateral DVTs indicate a systemic prothrombotic state, albeit they were relatively infrequent (Fig. 2).

Among the 675 DVT-positive patients, 344 (51.0%) had a single thrombus (only one vein involved) and 331 (49.0%) had multiple venous thrombi. In defining multiple thrombi, we counted involvement of multiple vein segments or both legs as “multiple.” The detection of nearly half the DVT patients with multiple clots underscores the potential for extensive thrombus burden even in distal veins.

### Thrombus multiplicity and anatomic distribution

Among patients with DVT, 344 of 675 (51.0%) had involvement of a single venous segment, and 331 of 675 (49.0%) had multiple segments involved (including bilateral events). In total, 1080 individual thrombi were recorded across all venous segments. Clots occurred predominantly in calf veins of the injured limb. The peroneal (fibular) veins were affected in 545 instances and the posterior tibial veins in 411 instances. Popliteal vein thrombosis was observed in 77 patients. Involvement of femoral segments was less frequent (superficial femoral 19, common femoral 16, profunda femoris 6), and iliac vein thrombosis was rare (external iliac 2, internal iliac 1, common iliac 1). Most popliteal and femoral events co-occurred with calf thrombosis rather than presenting in isolation. In the contralateral limb, DVT was uncommon and usually limited to the calf; for example, 45 peroneal vein clots were identified contralaterally, most in patients who also had ipsilateral thrombosis. Isolated contralateral DVT without any ipsilateral clot was recorded in 19 patients (2.8% of those with DVT) (Table 1).

**Table 1:** Distribution of thromboses in 675 DVT-positive patients (by vein segment and side)

Vein segment	Total clots (*n*)	Single-location clots	Part of multilocation clots	In injured leg	In contralateral leg
Peroneal veins	545	224	321	528	45
Posterior tibial veins	411	107	304	400	26
Popliteal vein	77	8	69	71	7
Superficial femoral vein	19	3	16	15	4
Common femoral vein	16	2	14	11	6
Deep femoral vein	6	0	6	3	3
Anterior tibial vein	2	0	2	2	0
External iliac vein	2	0	2	2	0
Internal iliac vein	1	0	1	0	1
Common iliac vein	1	0	1	1	0

### Pulmonary embolism and inferior vena cava filters

Temporary inferior vena cava (IVC) filters were placed in 86 patients (12.7% of those with DVT). Filter placement was considered selectively after consultation with vascular surgeons and multidisciplinary assessment, typically in patients with extensive proximal thrombosis (e.g., involving the iliac, common femoral, femoral/profunda femoris, or popliteal venous segments) and/or when therapeutic anticoagulation was contraindicated or needed to be temporarily withheld because of bleeding risk and perioperative planning. All filters were placed after DVT detection and prior to definitive fracture surgery. Symptomatic PE, confirmed by CTPA, occurred in five patients during hospitalization (0.15% of the total cohort; 0.74% among patients with DVT); all PE events were identified after DVT had been diagnosed. None of these five patients had an IVC filter in place prior to the PE event, and all survived after treatment (therapeutic anticoagulation with thrombolysis in two cases).

### Between-group comparisons of demographics and injury features (Table 2)

**Table 2:** Demographic data of patients with and without DVT

Demographics	Non-DVT group (*n* = 2691)	DVT group (*n* = 675)	*p*-Value
Age, years	45 (34–56)	50 (41–58)	< 0.001
Sex, *n* (%)			0.085
Male	1663 (61.8%)	442 (65.5%)	
Female	1028 (38.2%)	233 (34.5%)	
BMI, kg/m^2^	26.12 (24.97–26.73)	26.60 (24.49–27.42)	< 0.001
Time from injury to admission, days	2 (0–4)	0 (0–3)	< 0.001
Open fracture, *n* (%)			0.05
Yes	75 (2.8%)	31 (4.6%)	
No	2616 (97.2%)	644 (95.4%)	
Multiple injuries, *n* (%)			< 0.001
Yes	855 (31.8%)	372 (55.1%)	
No	1836 (68.2%)	303 (44.9%)	
Mechanism of injury, *n* (%)			0.602
I	431 (16.0%)	99 (14.7%)	
II	526 (19.5%)	140 (20.7%)	
III	1734 (64.4%)	436 (64.6%)	
Smoking history, *n* (%)			0.315
Yes	296 (11.0%)	66 (9.8%)	
No	2395 (89.0%)	609 (90.2%)	
Alcohol history, *n* (%)			< 0.001
Yes	262 (9.7%)	110 (16.3%)	
No	2429 (90.3%)	565 (83.7%)	

Values are presented as the number (%) or the median (interquartile range). A *p*-value < 0.05 was considered statistically significant

BMI, body mass index

Compared with patients without DVT, those with DVT were older (median 50 versus 45 years; *p* < 0.001) and had slightly higher BMI (≈ +0.5 kg/m^2^; *p* < 0.001). Sex distribution was similar (65.5% female versus 61.8% male; *p* = 0.085). Injury mechanism distributions were comparable between groups. Open fractures were marginally more frequent in DVT (4.6% versus 2.8%; *p* = 0.05). Multiple injuries were substantially more common with DVT (55.1% [372/675] versus 31.8% [855/2691]; *p* < 0.001). Alcohol use history was also more frequent in DVT (16.3% versus 9.7%; *p* < 0.001), whereas current smoking did not differ (≈10–11%; *p* = 0.315).

### Comorbid conditions (Table 3)

**Table 3:** Comorbidity data of patients with and without DVT

Comorbidities	Non-DVT group (*n* = 2691)	DVT group (*n* = 675)	*p*-Value
Coronary heart disease, *n* (%)			0.042
Yes	76 (2.8%)	30 (4.4%)	
No	2615 (97.2%)	645 (95.6%)	
Hypertension, *n* (%)			< 0.001
Yes	412 (15.3%)	147 (21.8%)	
No	2279 (84.7%)	528 (78.2%)	
Diabetes, *n* (%)			0.193
Yes	204 (7.6%)	62 (9.2%)	
No	2487 (92.4%)	613 (90.8%)	
Cerebral infarction, *n* (%)			0.357
Yes	68 (2.5%)	22 (3.3%)	
No	2623 (97.5%)	653 (96.7%)	
Anemia, *n* (%)			< 0.001
Yes	1342 (49.9%)	463 (68.6%)	
No	1349 (50.1%)	212 (31.4%)	
Hypoproteinemia, *n* (%)			< 0.001
Yes	277 (10.3%)	103 (15.3%)	
No	2414 (89.7%)	572 (84.7%)	
Hypokalemia, *n* (%)			0.003
Yes	313 (11.6%)	108 (16.0%)	
No	2378 (88.4%)	567 (84.0%)	
Hyponatremia, *n* (%)			< 0.001
Yes	141 (5.2%)	70 (10.4%)	
No	2550 (94.8%)	605 (89.6%)	
Arrhythmia, *n* (%)			0.011
Yes	80 (3.0%)	34 (5.0%)	
No	2611 (97.0%)	641 (95.0%)	
Hypomagnesemia, *n* (%)			0.839
Yes	200 (7.4%)	48 (7.1%)	
No	2491 (92.6%)	627 (92.9%)	
Hypophosphatemia, *n* (%)			0.091
Yes	147 (5.5%)	49 (7.3%)	
No	2544 (94.5%)	626 (92.7%)	
Hypocalcemia, *n* (%)			< 0.001
Yes	482 (17.9%)	178 (26.4%)	
No	2209 (82.1%)	497 (73.6%)	

Values are presented as the number (%) or the median (interquartile range). A *p*-value < 0.05 was considered statistically significant

Patients with DVT had higher prevalences of hypertension (21.8% versus 15.3%, *p* < 0.001), coronary heart disease (4.4% versus 2.8%, *p* = 0.042), anemia (68.6% versus 49.9%, *p* < 0.001), hypoproteinemia (15.3% versus 10.3%, *p* < 0.001), and electrolyte abnormalities (low sodium 10.4% versus 5.2%, *p* < 0.001; low potassium 16.0% versus 11.6%, *p* = 0.003; low calcium 26.4% versus 17.9%, *p* < 0.001). Documented arrhythmia was also more frequent (5.0% versus 3.0%, *p* = 0.011). Other comorbidities showed no significant differences (*p* > 0.05).

### Admission laboratory findings (Table 4)

**Table 4:** Laboratory results of patients with and without DVT

Laboratory results	Non-DVT group (*n* = 2691)	DVT group (*n* = 675)	*p*-Value
ALB, g/L	40.95 (38.53–43.66)	39.80 (37.02–42.60)	< 0.001
ALT, U/L	22.00 (14.00–33.50)	22.00 (15.00–34.71)	0.28
AST, U/L	21.00 (16.00–30.00)	22.00 (17.00–36.00)	< 0.001
AST/ALT	1.06 (0.74–1.33)	1.11 (0.81–1.44)	< 0.001
Ca, mmol/L	2.21 (2.13–2.30)	2.18 (2.08–2.27)	< 0.001
CHE, kU/L	7.89 (6.57–9.24)	7.46 (6.24–9.01)	< 0.001
CK, U/L	163.00 (80.05–489.17)	249.00 (113.10–655.79)	< 0.001
CK-MB, U/L	13.21 (9.70–21.00)	14.40 (10.50–22.31)	< 0.001
CL, mmol/L	104.44 (102.40–106.40)	104.27 (102.10–106.42)	0.254
CREA, μmol/L	62.63 (53.50–71.59)	63.13 (53.47–73.06)	0.096
DBIL, μmol/L	5.00 (3.57–6.54)	5.20 (3.50–7.08)	0.165
GAP, mmol/L	10.90 (8.90–12.90)	10.57 (8.65–12.64)	0.02
GLOB, g/L	23.63 (20.80–26.23)	23.10 (20.29–25.77)	0.011
GLU, mmol/L	5.74 (5.15–6.49)	6.04 (5.34–7.04)	< 0.001
HBDH, U/L	153.00 (129.98–172.00)	162.30 (137.06–188.00)	< 0.001
HDL-C, mmol/L	1.19 (1.01–1.33)	1.16 (0.98–1.33)	0.024
IBIL, μmol/L	11.20 (7.90–14.32)	11.50 (8.09–14.57)	0.12
K, mmol/L	3.92 (3.68–4.14)	3.89 (3.62–4.15)	0.145
LDH, U/L	195.77 (164.97–235.56)	204.17 (173.37–254.57)	< 0.001
LDL-C, mmol/L	2.58 (2.11–2.95)	2.59 (2.08–3.04)	0.479
Mg, mmol/L	0.89 (0.82–0.95)	0.88 (0.82–0.94)	0.23
Na, mmol/L	139.51 (137.90–141.20)	139.00 (137.00–140.80)	< 0.001
OSM, mOsm/kg	272.90 (268.30–278.60)	272.30 (267.70–278.05)	0.068
P, mmol/L	1.15 (1.00–1.28)	1.10 (0.97–1.25)	< 0.001
TBIL, μmol/L	16.26 (11.90–20.80)	17.12 (12.05–21.40)	0.077
TC, mmol/L	4.22 (3.64–4.75)	4.18 (3.58–4.79)	0.473
TCO2, mmol/L	24.76 (22.81–26.84)	24.89 (22.62–27.00)	0.989
TG, mmol/L	1.16 (0.81–1.55)	1.16 (0.85–1.57)	0.549
TP, g/L	64.40 (61.11–68.40)	62.99 (58.48–67.23)	< 0.001
UA, μmol/L	297.00 (229.00–358.00)	282.00 (218.00–345.50)	0.007
UREA, mmol/L	5.04 (4.11–6.00)	5.31 (4.25–6.42)	< 0.001
VLDL, mmol/L	0.53 (0.37–0.70)	0.53 (0.39–0.71)	0.509
BAS, ×10^9/L	0.03 (0.02–0.04)	0.03 (0.02–0.04)	0.587
EOS, ×10^9/L	0.07 (0.02–0.12)	0.06 (0.02–0.11)	0.008
HCT, %	36.79 (33.92–40.44)	34.93 (31.38–39.16)	< 0.001
HGB, g/L	123.67 (114.05–136.30)	117.50 (106.10–130.55)	< 0.001
IG, ×10^9/L	0.01 (0.00–0.01)	0.01 (0.00–0.01)	0.22
LYM, ×10^9/L	1.52 (1.16–1.85)	1.42 (1.08–1.75)	< 0.001
MCH, pg	30.85 (29.95–32.00)	30.99 (29.89–32.11)	0.455
MCHC, g/L	336.05 (330.40–341.80)	335.97 (330.10–342.10)	0.953
MCV, fL	91.75 (89.30–94.52)	92.11 (89.16–95.03)	0.249
MON, ×10^9/L	0.66 (0.49–0.81)	0.69 (0.51–0.88)	< 0.001
MPV, fL	8.68 (7.96–9.34)	8.59 (7.95–9.36)	0.613
NEU, ×10^9/L	5.93 (4.39–7.64)	6.74 (5.04–8.29)	< 0.001
PCT, %	0.19 (0.15–0.22)	0.18 (0.14–0.21)	0.005
PDW, fL	16.43 (16.04–16.87)	16.47 (16.00–16.91)	0.378
PLT, ×10^9/L	221.00 (177.65–248.00)	215.48 (168.05–239.25)	0.002
RBC, ×10^12/L	4.02 (3.70–4.40)	3.80 (3.44–4.23)	< 0.001
RDW-CV, %	13.19 (12.71–13.45)	13.20 (12.72–13.50)	0.083
WBC, ×10^9/L	8.65 (6.79–9.85)	9.42 (7.39–10.43)	< 0.001
APTT, s	30.30 (27.90–32.60)	29.20 (27.00–31.50)	< 0.001
APTT-R	0.95 (0.87–1.03)	0.93 (0.85–1.00)	< 0.001
AT III, %	98.47 (90.00–108.00)	97.63 (89.00–108.00)	0.152
D-dimer, mg/L FEU	1.31 (0.60–2.76)	2.10 (0.98–3.70)	< 0.001
FDP, mg/L	18.41 (5.91–18.41)	21.96 (10.55–21.96)	< 0.001
FIB, g/L	3.46 (2.81–4.09)	3.44 (2.76–4.22)	0.378
INR	1.06 (1.00–1.12)	1.06 (1.00–1.12)	0.703
PT, s	12.00 (11.30–12.70)	11.90 (11.20–12.70)	0.486
PTA, %	93.00 (85.00–101.00)	92.00 (85.00–101.00)	0.819
TT, s	14.30 (13.20–15.40)	14.10 (13.00–15.30)	0.084
TT-R	0.94 (0.89–0.97)	0.93 (0.88–0.97)	< 0.001

ALB, albumin; ALT, alanine aminotransferase; AST, aspartate aminotransferase; AST/ALT, AST-to-ALT ratio; Ca, calcium; CHE, cholinesterase; CK, creatine kinase; CK-MB, creatine kinase-MB; CL, chloride; CREA, creatinine; DBIL, direct bilirubin; GAP, anion gap; GLOB, globulin; GLU, glucose; HBDH, hydroxybutyrate dehydrogenase; HDL-C, high-density lipoprotein cholesterol; IBIL, indirect bilirubin; K, potassium; LDH, lactate dehydrogenase; LDL-C, low-density lipoprotein cholesterol; Mg, magnesium; Na, sodium; OSM, osmolality; P, phosphorus (inorganic phosphate); TBIL, total bilirubin; TC, total cholesterol; TCO^2^, total carbon dioxide; TG, triglycerides; TP, total protein; UA, uric acid; UREA, urea; VLDL, very-low-density lipoprotein (cholesterol); BAS, basophil; EOS, eosinophil; HCT, hematocrit; HGB, hemoglobin; IG, immature granulocytes; LYM, lymphocyte; MCH, mean corpuscular hemoglobin; MCHC, mean corpuscular hemoglobin concentration; MCV, mean corpuscular volume; MON, monocyte; MPV, mean platelet volume; NEU, neutrophil; PCT, plateletcrit; PDW, platelet distribution width; PLT, platelet count; RBC, red blood cell count; RDW-CV, red cell distribution width (coefficient of variation); WBC, white blood cell count; APTT, activated partial thromboplastin time; APTT-R, APTT ratio; AT III, antithrombin III; D-dimer, D-dimer; FDP, fibrin/fibrinogen degradation products; FIB, fibrinogen; INR, international normalized ratio; PT, prothrombin time; PTA, prothrombin activity; TT, thrombin time; TT-R, thrombin time ratio. *p* < 0.05 signifies statistical significance

Broadly, the DVT group demonstrated a pattern consistent with greater injury severity/inflammation and hypercoagulability: lower albumin and total protein (both *p* < 0.001); higher glucose (*p* < 0.001); modestly lower electrolytes (sodium, calcium, phosphorus; all *p* < 0.001); lower HGB/HCT/RBC with a small reduction in PLT (*p* ≤ 0.002); higher WBC with neutrophilia and monocytosis and lower lymphocytes (all *p* < 0.001); elevated injury/metabolic enzymes (CK, LDH, HBDH; all *p* < 0.001); shorter APTT (median 29.2 versus 30.3 s; *p* < 0.001); and higher D-dimer and FDP (both *p* < 0.001). Other chemistry, hematology, and coagulation indices were not significantly different (*p* > 0.05). To facilitate readability, detailed values are provided in Tables 2–4.

### Multivariable analysis of risk factors (Table 5)

**Table 5:** Binary logistic regression analysis of variables associated with DVT

Characteristics	OR	95% CI	*p*-Value
Multiple injuries	2.084	1.725–2.519	< 0.001
Age	1.022	1.013–1.03	< 0.001
Alcohol history	1.595	1.219–2.088	< 0.001
Anemia	1.542	1.175–2.023	0.002
APTT	0.932	0.887–0.979	0.005
Na	0.960	0.925–0.996	0.031

A *p*-value < 0.05 was considered statistically significant

APTT, activated partial thromboplastin time; Na, sodium

Table 5 presents the results of the multivariate logistic regression for DVT risk factors. Variables entered into the model included age, sex, BMI, injury-to-admission time, open fracture, multiple injuries, smoking, alcohol use, hypertension, coronary heart disease, anemia, hypoproteinemia, hyponatremia, APTT, D-dimer, and others, with *p* < 0.05 in univariate analysis. After stepwise selection, logistic regression identified six independent predictors of DVT: increasing age (per 1-year increase: OR = 1.022, 95% CI 1.013–1.030, *p* < 0.001), multiple injuries (OR = 2.084, 1.725–2.519, *p* < 0.001), anemia (OR = 1.542, 1.175–2.023, *p* = 0.002), history of alcohol use (OR = 1.595, 1.219–2.088, *p* < 0.001), and inverse associations for APTT (per 1-s increase: OR = 0.932, 0.887–0.979, *p* = 0.005) and serum sodium (per 1-mmol/L increase: OR = 0.960, 0.925–0.996, *p* = 0.031). Other covariates evaluated in the model were not independently associated after adjustment (all *p* > 0.05).

### Receiver operating characteristic (ROC) analysis (Fig. 3)

**Fig. 3:**
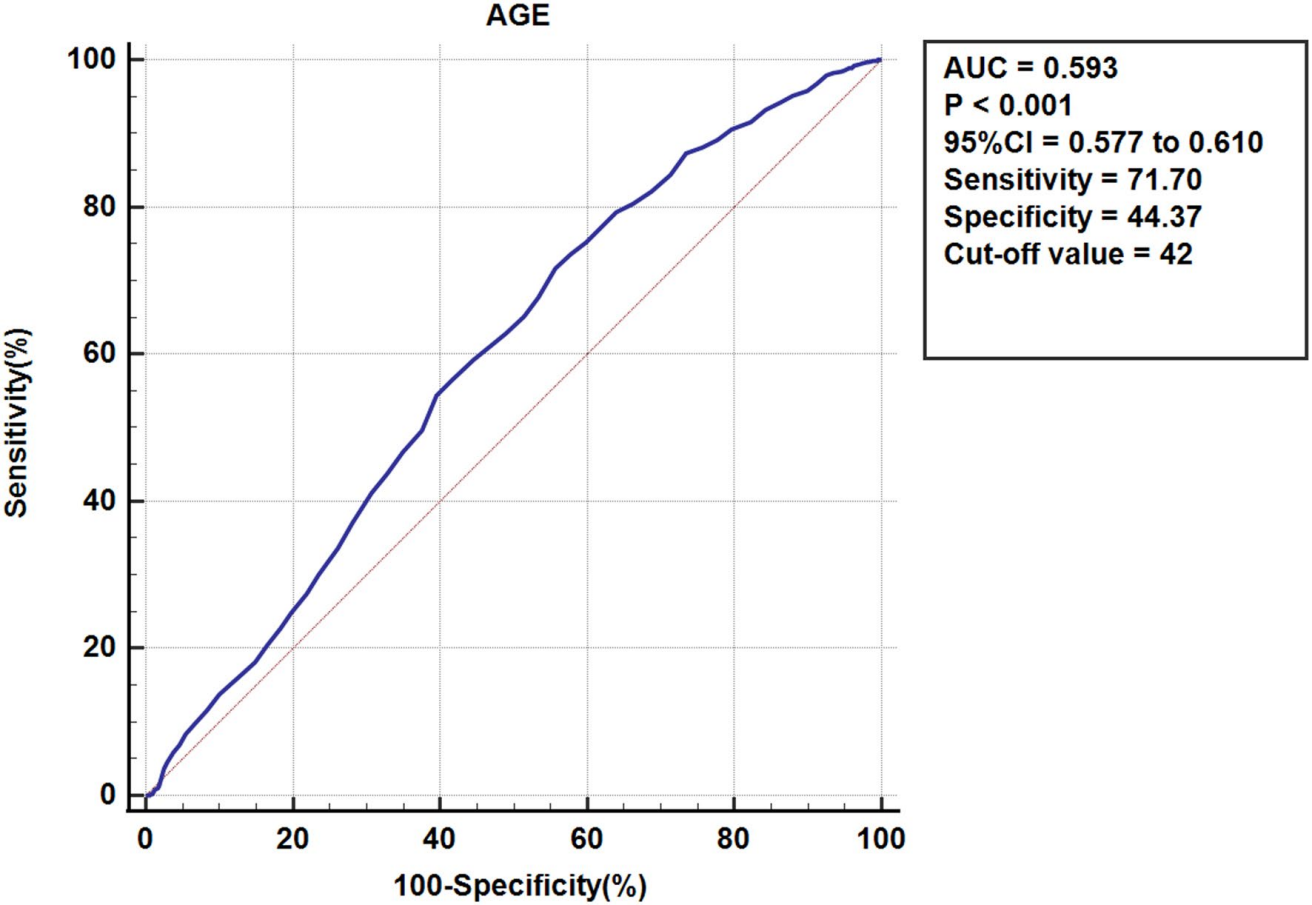
ROC curve for age

Receiver operating characteristic analysis of age alone showed modest discrimination for DVT (AUC = 0.593, 95% CI 0.577–0.610; *p* < 0.001). The Youden-derived cutoff was 42 years (positivity defined as age ≥ 42 years), yielding a sensitivity of 71.70% and a specificity of 44.37%. Age was modeled as a continuous variable in the multivariable analysis; therefore, the ROC-derived cutoff is provided for descriptive purposes and should not be interpreted as a definitive risk-stratification threshold.

## Discussion

### Main findings

In this large, multicenter cohort of patients with tibial plateau fractures, we observed an in-hospital DVT incidence of approximately 20.0%. About one in five patients with tibial plateau fractures developed a lower-extremity DVT during hospitalization. Notably, the vast majority of thrombi were located in the distal veins (below the knee), with relatively few proximal DVTs identified. Moreover, the thrombi were predominantly ipsilateral to the injured limb—DVT was confined to the fracture-affected leg in most cases, with only a small minority involving the contralateral leg. These findings underscore that tibial plateau fractures carry a high baseline risk of occult venous thrombosis throughout the index hospitalization, reinforcing the need for systematic surveillance and appropriate prophylaxis during the inpatient course.

We also identified several independent risk factors that predisposed patients to in-hospital DVT in this setting. Through multivariable logistic regression, increasing age emerged as a significant predictor—older patients were more likely to have DVT during hospitalization. Patients who sustained multiple injuries (polytrauma) in addition to the tibial plateau fracture also had higher odds of DVT, indicating that concomitant trauma increases thrombogenic risk. Among laboratory and clinical variables, anemia (lower hemoglobin) was associated with higher DVT incidence, as was a history of chronic alcohol use. Two other notable laboratory findings were a shorter APTT on admission and lower serum sodium level; both of these were independent risk factors for DVT in our cohort. Collectively, these six factors (increasing age, multiple injuries, anemia, alcohol use, shorter APTT, and low sodium) constituted the profile of a high-risk patient. To our knowledge, this is one of the first studies to examine such a comprehensive set of clinical and laboratory predictors in patients with tibial plateau fracture across two centers, with a sample size among the largest to date. These main findings allow for improved risk stratification and deepen our understanding of which patients are most vulnerable to venous thrombosis during the inpatient course.

### Comparison with previous literature

Our in-hospital DVT incidence (approximately 20%) falls within the mid-range of values reported for tibial plateau fractures (roughly 15–35% in cohorts using systematic ultrasound), with higher figures in studies that count calf muscle-vein thromboses (IMVT) and lower figures in symptom-triggered designs [12]. For example, Zhu et al. reported a 16.3% preoperative DVT incidence with routine ultrasound, and a smaller European cohort found approximately 23.6% despite standard prophylaxis [4, 13]; single-center series using especially sensitive protocols have reported rates approaching 30–40% [12, 14]. A key driver of variability is case definition: Many orthopedic series exclude IMVT from the primary DVT endpoint to enhance comparability, whereas others include it and consequently report higher rates [4, 15, 16]. In keeping with the former approach, our primary analysis excluded IMVT; were IMVT included, total in-hospital venous thrombosis in our cohort would rise from 675 to 1212 events (adding 537 isolated IMVT), approximately 36% of the sample. By contrast, clinically triggered imaging (without routine duplex) yields much lower rates (approximately 1–2%) because it may miss clinically silent distal thromboses that are detected by systematic surveillance [17]. Because symptom status at the time of DVT detection was not captured in a standardized manner, we did not classify events as symptomatic versus asymptomatic; the reported incidence reflects ultrasound-detected DVT identified through routine surveillance. Taken together, the 20% incidence we observed corroborates tibial plateau fractures as a high-risk injury for in-hospital VTE, exceeding general trauma estimates (≈0.4–11%) [18–20].

The anatomical distribution of thrombi in our patients also mirrors findings from comparable studies. We found that most DVTs were located in the calf veins (distal DVT), with far fewer clots in the popliteal or more proximal veins. This predominance of distal DVT is in line with other screening studies in orthopedic trauma—for instance, Zhu et al. observed distal DVTs in approximately 15% of patients but proximal DVTs in only approximately 1% [4]. Hermel et al. likewise noted that the distal veins (particularly the fibular veins) were most frequently affected in patients with tibial plateau fracture [13]. From a clinical standpoint, proximal DVT generally carries a higher risk of embolization and is more likely to warrant urgent therapeutic anticoagulation and closer monitoring, whereas isolated distal DVT—particularly when detected through routine screening—often has a lower short-term embolic risk and may be managed with therapeutic anticoagulation or structured serial imaging depending on thrombus extent, symptoms, and bleeding risk. Accordingly, the high proportion of distal events in our cohort should be interpreted in the context of systematic duplex surveillance, which is sensitive to clinically silent infrapopliteal thrombosis. In addition, we excluded isolated intermuscular calf-muscle vein thrombosis (soleal/gastrocnemius) from the primary endpoint; studies that include these events typically report higher “distal DVT” rates, which should be considered when comparing incidence across cohorts. Importantly, our data confirm that thromboses overwhelmingly occurred in the injured limb (ipsilateral to the fracture), supporting the concept that local trauma-related factors (soft tissue injury, venous stasis from immobilization) play dominant roles in precipitating clots in the affected leg [4].

In our research, increasing age as a risk factor is well documented in orthopedic thromboprophylaxis research. Consistent with our results, multiple studies have found that older patients face higher odds of thrombosis after fractures [21–23]. Age likely reflects the accumulation of prothrombotic tendencies and comorbidities in older individuals, and our findings reinforce its importance. We did not find a significant sex difference in DVT risk, whereas some previous studies have. Zhu et al. noted a higher DVT rate in males, and a recent series of clinically important VTE in tibial plateau fractures found male sex to be an independent risk factor (with an 11-fold higher risk of symptomatic events in males) [4, 17]. This discrepancy likely reflects different endpoints (screen-detected versus clinically overt events) and cohort differences; when both sexes undergo uniform screening, sex effects may attenuate.

Our finding that multiple injuries (polytrauma) are associated with greater DVT risk is strongly supported by prior research [7, 12, 17, 24]. Tibial plateau fractures often result from high-energy mechanisms, and patients with additional fractures or injuries are known to have elevated thrombotic risk. A recent study in *Scientific Reports* identified concomitant spine injuries and other extremity fractures as independent predictors of VTE in patients with tibial plateau fracture [17]. Those authors reported an odds ratio of 9.5 for VTE with associated spine injury, presumably because such severe injuries often delay mobilization and chemoprophylaxis. Similarly, Stawicki et al. found that multiply injured patients had higher DVT rates than those with isolated limb fractures [25]. Our results concur: Patients with additional traumatic injuries (e.g., ipsilateral limb injuries or other system injuries) were at markedly higher risk for DVT than those with an isolated plateau fracture. This highlights the additive effect of injury burden—greater tissue trauma, prolonged immobilization, and staged procedures likely promote venous stasis and clot formation.

Several hematological and biochemical factors emerged as significant predictors, aligning with and expanding upon previous literature. One such factor is anemia, which we found to be independently associated with DVT, consistent with observations in other orthopedic populations [26, 27]. Anemia may index blood loss, inflammation, or physiologic stress—each plausibly augmenting thrombogenic potential. Another key finding was that a shorter APTT was independently associated with DVT; accumulating evidence indicates that reduced APTT reflects accelerated intrinsic-pathway activity and a hypercoagulable state [28, 29]. Tripodi et al. showed that a shortened baseline APTT is an independent risk marker for VTE [28, 29]. Our two-center cohort adds trauma-specific evidence that a short admission APTT—a widely available test—has risk-stratification value. We additionally identified lower serum sodium as an independent correlate, echoing Zhu et al.’s report in patients with tibial plateau fracture [4] and observational links between dysnatremia and thrombotic risk in other settings [30, 31]. Finally, chronic alcohol use was associated with higher DVT odds, consistent with large trauma datasets and population studies [24, 32]; this supports considering alcohol history when gauging VTE risk in orthopedic trauma. In summary, our findings both corroborate many risk factors previously reported—such as increasing age, injury severity (polytrauma), and chronic alcohol use—and highlight less well-recognized factors such as anemia, shortened APTT, and lower serum sodium level. The convergence of evidence from our study and others points to a multifactorial thrombogenic process in patients with tibial plateau fracture, where patient comorbidities, injury characteristics, and laboratory biomarkers all contribute to risk. Each piece of this risk profile is valuable for building a comprehensive prediction and prevention strategy, as discussed below.

### Potential mechanisms

The high incidence of DVT in the preoperative period following tibial plateau fractures can be explained by classic thrombogenic mechanisms. Tibial plateau fractures often result in prolonged limb immobilization while awaiting definitive fixation—patients may be placed in external fixation or splints for days to weeks to allow soft tissue swelling to subside. This immobilization leads to venous stasis in the injured limb, fulfilling one element of Virchow’s triad (stasis) for thrombosis [13]. In addition, the trauma itself causes local endothelial injury in vessels and a systemic inflammatory response that promotes a hypercoagulable state. Major fractures are known to activate the coagulation cascade through tissue factor release and cytokine-mediated increases in clotting factors. Indeed, trauma patients can exhibit a so-called procoagulopathy shortly after injury—characterized by elevated fibrinogen and factor VIII levels and suppressed fibrinolysis—which predisposes to fibrin clot formation [5, 33]. In our cohort, 64.0% of DVTs were first detected preoperatively, supporting that many thrombi are identified early during the inpatient course. This pattern is consistent with prior reports suggesting that DVT can be detected within the first few days after injury (e.g., Zhu et al. reported a median of 2 days to DVT detection) [4]. This timing implicates the acute post-injury phase as critical for clot development. Therefore, the mechanism of preoperative DVT in tibial plateau fractures likely involves a convergence of stasis (due to limb immobilization and bed rest), endothelial damage (due to fracture and potential surgical manipulations such as traction or reduction maneuvers), and hypercoagulability (due to the trauma-induced coagulation response). Each of these factors aligns with Virchow’s triad, and they collectively dramatically elevate the risk of venous thrombosis.

The distal predominance and ipsilateral localization of DVT in these patients also have mechanistic underpinnings. Distal (calf) veins are smaller and more susceptible to stasis when muscle pumping is eliminated by splinting or external fixation. The calf muscle inactivity leads to reduced venous return, and the injured leg often remains dependent (hanging down) when the patient sits, further slowing venous flow. This likely explains why calf vein thrombi are so common. Furthermore, tibial plateau fractures can involve significant soft tissue injury around the knee; internal damage or inflammation near the popliteal fossa might activate the coagulation cascade locally. However, if a clot initiates in the calf veins, it may remain confined distally unless it propagates proximally over time. The fact that we and others detected relatively few popliteal or femoral DVTs preoperatively suggests that either proximal clots are less common or that routine thromboprophylaxis and systematic screening catch clots while they are still distal [4, 13]. In this context, routine thromboprophylaxis (early LMWH in eligible patients) may have limited proximal propagation and clinically significant embolic events, which could partly explain the low proportion of proximal DVT and the rarity of PE. In polytrauma or staged-procedure cases, LMWH could be deferred or temporarily interrupted because of bleeding risk, and was resumed once hemostasis was secured, although these delays were not quantified in detail. The ipsilateral predominance is expected because the injured limb experiences the brunt of the risk factors: It is the limb that is immobilized, often elevated or in traction, and it has local trauma-related vascular injury and inflammation. The uninjured leg, by contrast, remains mobile (the patient can often exercise or at least move the other leg) and lacks direct injury, making thrombosis there less likely. Only in cases with significant systemic hypercoagulability or prolonged bed rest without moving either leg would the contralateral limb be at substantial risk, which matches the low contralateral DVT rates observed.

Regarding the specific risk factors we identified, each can be linked to plausible biological mechanisms for thrombosis:

1. Increasing age: Patients with increasing age often have reduced venous valve competence and slower venous return in the legs, contributing to stasis. Aging is also accompanied by elevated baseline levels of procoagulant factors (e.g., factor VIII and fibrinogen tend to increase with age) and reduced fibrinolytic activity. Comorbidities common in older adults (such as malignancy or cardiovascular disease) can heighten coagulation activation as well. Collectively, these changes create a prothrombotic milieu in elderly patients [34, 35]. Thus, an older patient with tibial plateau fracture is inherently more prone to clotting than a younger, healthier patient under the same conditions of injury and immobilization.
2. Multiple injuries (polytrauma): When a tibial plateau fracture is part of a multiple-injury complex (for example, concurrent long-bone fractures, spinal injuries, or internal organ injuries), the systemic inflammatory response is amplified. Polytrauma triggers a “second hit” of coagulation activation through extensive tissue damage. Additionally, patients with multiple injuries often require intensive care (including ventilation, central lines, etc.), prolonged bed rest, and sometimes staged surgeries—all factors that exacerbate stasis and hypercoagulability [33]. Therefore, the presence of multiple injuries synergizes with the fracture to heighten thrombotic risk via increased coagulation drive.
3. Anemia: The link between anemia and thrombosis is an intriguing one that is still being elucidated. One hypothesis revolves around the role of red blood cells (RBCs) in thrombosis. High hematocrit (polycythemia) is known to increase blood viscosity and promote stasis, yet paradoxically anemia (low hematocrit) has also been associated with VTE [36, 37]. In anemia, the reduced RBC count might lead to a relative excess of platelets and procoagulant factors per unit volume of blood, and turbulent flow patterns can arise in anemia that favor clotting. Severe anemia may also reflect significant blood loss and tissue injury, which can induce a compensatory inflammatory response and hypercoagulability [38]. It suggests that hemoglobin could serve as a surrogate for injury severity or a physiologic trigger for thrombosis (through mechanisms such as tissue hypoxia and compensatory erythropoiesis, which can activate platelets and coagulation).
4. Alcohol use: Chronic alcohol use is linked to prothrombotic change via liver-related hemostatic imbalance: patients with chronic liver disease show elevated von Willebrand factor (vWF) and factor VIII with reduced protein C, shifting toward hypercoagulability [39]. Alcohol-related dehydration (often compounded by poor nutrition) may raise viscosity and reduce flow, potentially augmenting VTE risk. Dehydration has been independently associated with VTE in hospitalized patients [40]. Behaviorally, alcohol use correlates with high-energy trauma exposure. Clinically, alcohol use independently predicted DVT after orthopedic trauma in a American College of Surgeons National Surgical Quality Improvement Program  analysis (approximately 56,000 patients; ≈threefold higher odds), and a nationwide claims study found higher subsequent VTE after alcohol intoxication [24]. Together, these data support our finding that a history of alcohol use identifies a subgroup with heightened thrombotic susceptibility during hospitalization.
5. Short APTT (hypercoagulability): Short APTT reflects accelerated intrinsic-pathway activity and a hypercoagulable shift. After injury, acute-phase rises in factor VIII and fibrinogen can further shorten APTT, marking early procoagulant activation [29, 35, 41]. Case–control and cohort data consistently link shortened APTT with venous thromboembolism: In 605 cases versus 1290 controls, short APTT was associated with higher odds of first-ever VTE; in a population cohort, APTT below the median doubled the 13-year risk of incident VTE; and after anticoagulation withdrawal, abnormally short APTT predicted recurrent VTE [28, 41, 42]. Taken together, these findings support using admission APTT as a pragmatic early marker to flag trauma patients at increased thrombotic risk and to consider intensified thromboprophylaxis when clinically appropriate.
6. Low serum sodium level: In trauma, a low serum sodium level often reflects physiologic stress rather than a direct coagulation effect: Non-osmotic arginine vasopressin release (syndrome of inappropriate antidiuresis) promotes water retention and dilutional sodium lowering [43]. Sodium imbalance has been linked to venous thromboembolism risk in cohort studies [31]. Consistent with this framework, tibial plateau fracture cohorts—ours included—have identified lower admission sodium as an independent correlate of in-hospital deep vein thrombosis, suggesting that a low sodium level flags a high-stress, immobilized phenotype prone to clot formation [4]. Clinically, when a trauma patient presents with low sodium—particularly alongside anemia or multiple injuries—heightened DVT surveillance and optimization of fluids/nutrition are warranted. More research is needed on the pathophysiological connections, but clinicians should be mindful that a trauma patient with hyponatremia may not only be medically complex but also thrombotically at risk.

In essence, each risk factor we identified can be mapped to one or more elements of the thrombotic triad or trauma pathophysiology. Older, sicker patients (with anemia or hyponatremia) have more fragile homeostasis and are quick to develop clots under stress. More severely injured patients (polytrauma, high-energy fractures) generate stronger procoagulant signals and suffer longer immobilization. Moreover, baseline hypercoagulability (short APTT, possibly influenced by genetic or acquired factors, and lifestyle factors such as alcohol) sets the stage for thrombosis even before injury occurs. The convergence of these factors in a given patient explains why some patients with tibial plateau fracture develop DVT within days while others do not. Understanding these mechanisms provides a biological rationale for our risk-factor findings and points toward targeted preventive strategies.

## Clinical implications

Given the approximately 20% in-hospital DVT burden, systematic or risk-adapted duplex ultrasonography may be considered, particularly in patients with multiple risk factors. Thromboprophylaxis should follow contemporary guidelines, and patients with higher predicted risk may merit closer monitoring and individualized perioperative management. Prospective studies are needed to determine the optimal screening strategy and whether risk-tailored intensification improves clinical outcomes. While LMWH remains foundational, patients with multiple risk factors (increasing age, polytrauma, anemia, low sodium, short APTT, alcohol use) may be considered for earlier initiation and/or closer monitoring of guideline-concordant chemoprophylaxis, and adjunct mechanical measures when feasible, with closer surveillance. Consistent with contemporary guidelines [9, 10], temporary IVC filters may be considered selectively when anticoagulation is contraindicated or must be temporarily withheld around urgent fixation, after multidisciplinary assessment. In our cohort, filters were placed after DVT detection and before definitive surgery, underscoring the need for prospective validation of risk-adapted screening and perioperative management strategies.

## Limitations

Despite the comprehensive analysis and robust methodology of our study, several limitations warrant mention. Firstly, this was a retrospective analysis; associations cannot establish causality, and residual confounding likely remains despite multivariable adjustment, including unmeasured factors such as perioperative mobility, bleeding severity, and the exact timing/dose adherence of thromboprophylaxis. We lacked several covariates (e.g., inherited/acquired thrombophilias, peri-injury hormonal status). Secondly, we could not stratify by tibial plateau fracture classification (e.g., Schatzker or Arbeitsgemeinschaft für Osteosynthesefragen/Orthopaedic Trauma Association [AO/OTA]) because standardized fracture typing was not consistently recorded in the registry/operative notes across the study period, and a uniform retrospective reclassification from imaging was not feasible. Given that fracture morphology and soft tissue severity may influence immobilization, surgical timing/strategy, and mobilization, this may contribute to residual confounding in DVT risk estimates. Thirdly, although thromboprophylaxis followed an institutional protocol, the exact timing, dosing adjustments, interruptions/continuity of LMWH, and the duration/feasibility of mechanical prophylaxis were not uniformly documented and could not be fully quantified in this retrospective dataset; these variations may have influenced in-hospital DVT risk and contributed to residual confounding. Fourthly, our primary endpoint excluded intermuscular calf-muscle vein thrombosis (soleal/gastrocnemius). Cohorts that include these events report higher rates, which may limit direct cross-study comparisons. Fifthly, symptom status at the time of DVT detection was not captured in a standardized manner; therefore, we did not classify DVT as symptomatic versus asymptomatic, and all ultrasound-detected events were analyzed together. This may limit comparisons with studies reporting symptomatic DVT only. Sixthly, We did not have standardized injury severity scores (ISS/AIS); thus, our operational definition of multiple injuries may limit comparability with studies defining polytrauma by ISS thresholds. Seventhly, The exact time of injury onset was not consistently recorded with sufficient granularity; thus, we were unable to reliably analyze time from injury to first DVT detection.

Most importantly, follow-up ended at hospital discharge; therefore, we could not assess post-discharge (delayed) DVT or PE, thrombus progression/recurrence, or longer-term sequelae such as post-thrombotic syndrome, and the reported incidence should be interpreted as the cumulative in-hospital, ultrasound-detected DVT burden. Last but not least, although several independent predictors were identified, DVT is multifactorial, and model discrimination is inherently limited; interactions among variables (e.g., alcohol use, anemia, low serum sodium) may persist. Prospective, multicenter studies with standardized prophylaxis, detailed fracture classification, and longitudinal follow-up are warranted.

## Conclusions

In this large cohort of tibial plateau fractures, the in-hospital incidence of lower-extremity DVT was high (approximately 20%), predominantly distal, and ipsilateral. Independent correlates of DVT included increasing age (with only modest discrimination when used alone; AUC 0.593), multiple injuries, anemia, history of alcohol use, and inverse associations with APTT (shorter times) and serum sodium (lower levels). These readily obtainable variables may help inform inpatient risk stratification and the consideration of systematic or risk-adapted duplex surveillance alongside guideline-concordant thromboprophylaxis. Prospective multicenter studies are needed to validate these predictors and to determine whether risk-tailored surveillance and perioperative management improve clinically meaningful outcomes.

## Data Availability

Yes.

## Abbreviations

DVT: Deep vein thrombosis
PE: Pulmonary embolism
VTE: Venous thromboembolism
LMWH: Low-molecular-weight heparin
IVC: Inferior vena cava
APTT: Activated partial thromboplastin time
PT: Prothrombin time
INR: International normalized ratio
TT: Thrombin time
TT-R: Thrombin time ratio
PTA: Prothrombin time activity
FDP: Fibrin/fibrinogen degradation products
AT III: Antithrombin III
LDH: Lactate dehydrogenase
HBDH: Hydroxybutyrate dehydrogenase
CK: Creatine kinase
CK-MB: Creatine kinase-MB
ALB: Albumin
TP: Total protein
GLOB: Globulin
HDL-C: High-density lipoprotein cholesterol
LDL-C: Low-density lipoprotein cholesterol
TC: Total cholesterol
TG: Triglycerides
Na: Sodium
K: Potassium
Cl: Chloride
Ca: Calcium
Mg: Magnesium
WBC: White blood cell
RBC: Red blood cell
HGB: Hemoglobin
HCT: Hematocrit
PLT: Platelet count
MPV: Mean platelet volume
PDW: Platelet distribution width
PCT: Plateletcrit
MCV: Mean corpuscular volume
MCH: Mean corpuscular hemoglobin
MCHC: Mean corpuscular hemoglobin concentration
RDW: Red cell distribution width
TBIL: Total bilirubin
DBIL: Direct bilirubin
ALT: Alanine aminotransferase
AST: Aspartate aminotransferase
ALP: Alkaline phosphatase
GGT: Gamma-glutamyl transferase
BMI: Body mass index
IQR: Interquartile range
OR: Odds ratio
CI: Confidence interval
ROC: Receiver operating characteristic
AUC: Area under the curve
MDT: Multidisciplinary team
IMVT: Intermuscular calf-muscle vein thrombosis
CBC: Complete blood count
SC: Subcutaneous
q12h: Every 12 h
IU: International unit
anti-Xa: Anti-factor Xa
AO/OTA: Arbeitsgemeinschaft für Osteosynthesefragen/Orthopaedic Trauma Association
CTPA: Computed tomography pulmonary angiography
